# Towards Goals to Refine Prophylactic and Therapeutic Strategies Against COVID-19 Linked to Aging and Metabolic Syndrome

**DOI:** 10.3390/cells10061412

**Published:** 2021-06-06

**Authors:** Chong-Hyun Shin, Ki-Hye Kim, Subbiah Jeeva, Sang-Moo Kang

**Affiliations:** Center for Inflammation, Immunity & Infection, Institute for Biomedical Sciences, Georgia State University, Atlanta, GA 30303, USA; kkim39@gsu.edu (K.-H.K.); jsubbiah@gsu.edu (S.J.)

**Keywords:** COVID-19, SARS-CoV-2, aging, metabolic syndrome, obesity, diabetes, vaccines, therapeutic drugs

## Abstract

The severe acute respiratory syndrome coronavirus 2 (SARS-CoV-2) gave rise to the coronavirus disease 2019 (COVID-19) pandemic. A strong correlation has been demonstrated between worse COVID-19 outcomes, aging, and metabolic syndrome (MetS), which is primarily derived from obesity-induced systemic chronic low-grade inflammation with numerous complications, including type 2 diabetes mellitus (T2DM). The majority of COVID-19 deaths occurs in people over the age of 65. Individuals with MetS are inclined to manifest adverse disease consequences and mortality from COVID-19. In this review, we examine the prevalence and molecular mechanisms underlying enhanced risk of COVID-19 in elderly people and individuals with MetS. Subsequently, we discuss current progresses in treating COVID-19, including the development of new COVID-19 vaccines and antivirals, towards goals to elaborate prophylactic and therapeutic treatment options in this vulnerable population.

## 1. Introduction

The severe acute respiratory syndrome coronavirus 2 (SARS-CoV-2) brought about the coronavirus disease 2019 (COVID-19) pandemic causing an unprecedented crisis worldwide. SARS-CoV-2 is conveyed primarily through respiratory droplets [1]; SARS-CoV-2 enters the host airway epithelial cells via binding to the receptor, angiotensin-converting enzyme 2 (ACE2) [2]. While SARS-CoV-2 leads to a mild to moderate symptoms in most people, it provokes severe complications, including pneumonia, acute respiratory distress syndrome (ARDS), acute kidney injury, and organ failure with subsequent mortality, in older individuals and people with underlying medical conditions of heart/lung disease or diabetes mellitus (DM).

Aging leads to a loss of physiological integrity, functional decline, and increased vulnerability to death [3]. The prevalent feature of aging and age-related diseases is chronic inflammation, known as “inflammaging”; it is the low-grade, systemic inflammation without overt infection, and is a key risk factor for morbidity and mortality in older individuals [4]. The major contributing factors of inflammaging are: (1) endogenous host-derived cell debris that accumulate with age [5]; (2) senescent cells and their senescence-associated secretory phenotype (SASP) [6]; (3) immunosenescence exacerbated by continuing infections of viruses, leading to mild hyperactivity of innate immunity [7,8]; (4) gut- and other microbiota-derived harmful products and metabolites [9]; and (5) increasing activation of coagulation system with aging [5]. Specifically, constant activation of intrinsic receptors by endogenous signals has been suggested to direct a chronic state of background inflammation [7,8]. Cellular senescence and the attainment of the SASP by fibroblasts, endothelial, and immune cells have also been suggested to bestow inflammaging; cell senescence triggers the deposition of dysfunctional, terminally differentiated B, T, and natural killer (NK) cells [6]. Notably, inflammaging mechanistically connects aging/age-associated diseases with metabolic syndrome (MetS) [10].

MetS is a constellation of conditions, such as abdominal obesity, high blood pressure, insulin resistance (IR)/glucose intolerance, atherogenic dyslipidemia, pro-thrombotic state, and pro-inflammatory state; the likelihood of heart disease, stroke, and type 2 diabetes mellitus (T2DM) is increased by MetS [11]. Clinically, a diagnosis of MetS is determined by the presence of 3 or more of the following factors: increased waist circumference (>102 cm (>40 in) in men; >88 cm (>35 in) in women), hypertriglyceridemia (≥150 mg/dL), increased blood pressure (systolic ≥ 130 and/or diastolic ≥ 85 mmHg), decreased high-density lipoprotein (HDL) cholesterol (<40 mg/dL in males; <50 mg/dL in females), and dysglycemia (≥100 mg/dL) (Adult Treatment Panel III report (ATP III), National Cholesterol Education Program) [11].

The obesity epidemic has been crucially noticed as over 1.9 billion people being overweight with the worldwide prevalence of obesity nearly tripled since 1975 [12]. Obesity increases the risk of premature death by 1.45- to 2.76-fold and shortens lifespan by up to 20 years [13,14]. Obesity-induced systemic chronic low-grade inflammation is an essential mechanistic cause of MetS [12]. It is distinct from other inflammatory prototypes as it involves in continuous activation of the innate immune system, leading to tissue remodeling and systemic metabolic deterioration over time; it also sets off maladaptive reactions, resulting in substantial tissue damage. Multiple organs, including brain, heart, insulin-sensitive tissues (adipose, liver, and skeletal muscle), and pancreas are sites of inflammation, disrupting glucose and energy metabolism [12]. Accordingly, obesity and aging share a similar spectrum of phenotypes and mechanisms, such as redox imbalance, mitochondrial dysfunction, accumulation of macromolecules, weakened immunity, and systemic inflammation, and are sources of age-related diseases [15]. Unsurprisingly, conditions and comorbidities of aging and age-related diseases mirror those of obesity [15].

The essential influence of aging and MetS on viral infection and host susceptibility has been an important research focus [16,17,18,19]. Viruses can reprogram the host cell metabolism to allow their replication and progeny release while averting host immune responses to evade pathogen recognition. Because aging and metabolic disorders debilitate immune system, viral infection further impairs immune responses and enhances severity of metabolic disease [16,17,18,19]. Consequently, elderly people and patients with MetS often experience worse progression of viral diseases, such as unparalleled severe outcomes of COVID-19 (over 3 million death worldwide).

In this review, we examine the prevalence and molecular mechanisms underlying increased risk of COVID-19 in elderly people and people with MetS. Additionally, we discuss current progresses in treating COVID-19 with focuses on vaccine development and antivirals, reinforcing therapeutic approaches to surmount the pandemic.

## 2. Possible Molecular Mechanisms Underlying Increased Risk of COVID-19 Complications Associated with Aging and MetS

### 2.1. Aging and COVID-19

The majority of deaths caused by COVID-19 has been occurred in people over the age of 65. Age is thus clearly the most notable risk factor for COVID-19 mortality [20]. Similarly, older people are disproportionately affected by human coronaviruses and influenza viruses [21], yet except vaccines, no apparent therapeutic strategies have been suggested to protect this fraction of the population. A study in the UK (109,000 randomly selected teenagers and adults) showed that the infection fatality ratio (IFR) was close to zero (15–44-year-olds), whereas increasing to 3.1% (65–74-year-olds) and to 11.6% (older than 74 years) [22]. A comparable tendency was observed in another study from Spain (61,000 randomly selected residents); the overall IFR was close to zero (under 50 years old), whereas 11.6% for men and 4.6% for women (80 years old and over) [23]. Importantly, the age-specific COVID-19-associated death data (45 countries and 22 seroprevalence studies) indicated that spreading the virus in nursing homes or elderly-care facilities appears to be an essential factor [24]. 

**Figure 1:**
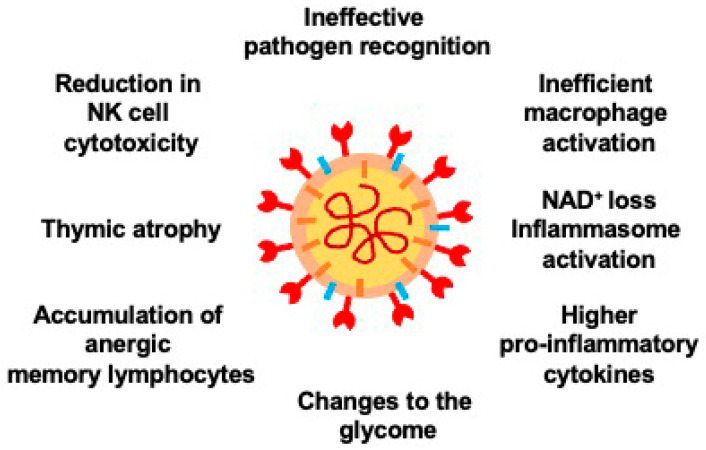
Schematic diagram of how age-related alterations escalate susceptibility of COVID-19. The aging immune system are subject to innate and adaptive immunosenescence, and inflammaging. An age-associated decrease in NAD^+^ leads to de-repression of NLRP3 and activation of inflammasome in older people, further aggravating the cytokine storm. The glycome regulating a variety of immune signaling pathways changes during aging.

Age-related changes of the immune system play critical roles in bestowing COVID-19 susceptibility: impair the body’s ability to carry away the SARS-CoV-2 virus and trigger cytokine storms, causing disseminated intervascular coagulation (DIC) and multi-organ injury as well as failure (Figure 1) [25]. The aging immune system undergoes immunosenescence, a progressive diminution in immune function [25]. The other representative immune system change during aging is inflammaging, a persisting elevation in systemic inflammation [4]. 

Within immunosenescence, there are: 1) innate immunosenescence, including inefficient recognition of pathogens and macrophage activation, and a decrease in natural killer (NK) cell cytotoxicity, and 2) adaptive immunosenescence, such as thymic degeneration and deposition of anergic memory lymphocytes [25]. Alveolar macrophages (AMs) are phagocytes that reside in the pulmonary alveoli. AMs display impaired function during aging; significant reduction of their conversion potential between pro- and anti-inflammatory phenotype [26]; downregulation of Toll-like receptor (TLR) expression and downstream signaling as well as pro-inflammatory and immunomodulatory cytokine production [8]. In older individuals, AMs exhibit diminished capacity to become pro-inflammatory state upon encountering pathogen, thus, spurring COVID-19 in its early stages, whereas AMs are correlated with the excessive lung damage in advanced stages of COVID-19 [25]. Moreover, severe COVID-19 patients had less expanded bronchoalveolar CD8^+^ T cells [27]; reduction and functional exhaustion of T cells were also observed in COVID-19 patients with high levels of the immune-exhaustion marker programmed cell death-1 (PD-1) in peripheral blood T cells [28].

Multiple evidence implicates that the cytokine storm, an uncontrollable inflammatory response by the immune system [29], is primarily derived from inflammaging, exacerbated by obesity and microbial dysbiosis [30,31]. In rodent animal models, higher risk of cytokine storm syndrome is closely correlated with inflammaging [32]; in humans, excessive basal circulating levels of pro-inflammatory cytokines, including interleukin (IL)-6, tumor necrosis factor (TNF)-α, IL-1α and C-reactive protein (CRP), are associated with age [33,34]. An age-associated decline in nicotinamide adenine dinucleotide (NAD^+^) results in de-repression of nucleotide-binding domain (NOD)-like receptor protein 3 (NLRP3), the major protein component of the inflammasome [35,36,37,38]. It was shown that SARS-CoV-2 proteins enhance the activity of poly-ADP-ribose polymerases (PARPs) PARP9, -10, -12, and -14, and decrease NAD^+^ levels [39]. Thus, it is likely that the decline of NAD^+^ levels, aggravated by COVID-19, increases the activity of NLRP3 and triggers cytokine storms in COVID-19 patients; restoring normal NAD^+^ levels likely alleviates COVID-19 symptoms.

Aging changes the glycome through non-enzymatic glycation; after exposure to sugars, proteins and lipids become glycated and develop advanced glycation end products (AGEs) [40]. AGEs form in hyperglycemic conditions and contribute to the pathology of many age-related, metabolic diseases and oxidative stress as well as inflammation [41]. The NLRP3 inflammasome activation in lipopolysaccharide-primed macrophages was shown to be induced by SARS-CoV 3a [42]; AGEs may escalate COVID-19 severity in elderly people by hindering NLRP3 inflammasome-mediated innate immune responses during the early stages of viral infection [43]. Furthermore, DIC seen in COVID-19 patients is likely to be triggered by AGEs considering their active role in pro-coagulation pathways [41].

Increased age is clearly the key risk factor for COVID-19 mortality [20]. Accordingly, as prophylactic approaches, not only vaccination but also activation of the body’s defenses against aging by using geroprotectors are in consideration. Low dose mechanistic target of rapamycin (mTOR) inhibitors was shown to reduce rates of infection and improved immunity and response to influenza vaccination in elderly [44,45]; consistently, metformin that inhibits the mTOR pathway and activates 5′-AMP-activated protein kinase (AMPK) as well as lowers blood glucose levels has been proposed to hamper severe SARS-CoV-2 infection in elderly [46].

### 2.2. Obesity and COVID-19

Multiple studies suggest that enhanced body mass index (BMI) is a critical indicator of adverse diagnosis of COVID-19 patients [47]. The data from Shenzhen, China demonstrated that obese and overweight patients were susceptible to acquire severe pneumonia compared with normal-weight patients [48]; a study in France described that obesity (BMI > 35 kg/m^2^) escalated the risk for ventilation [49]; another study from the United Kingdom, obesity was a risk factor for mortality with a significant BMI gradient [50]. These studies indicate that obesity is strongly correlated with severe COVID-19 and death.

**Figure 2:**
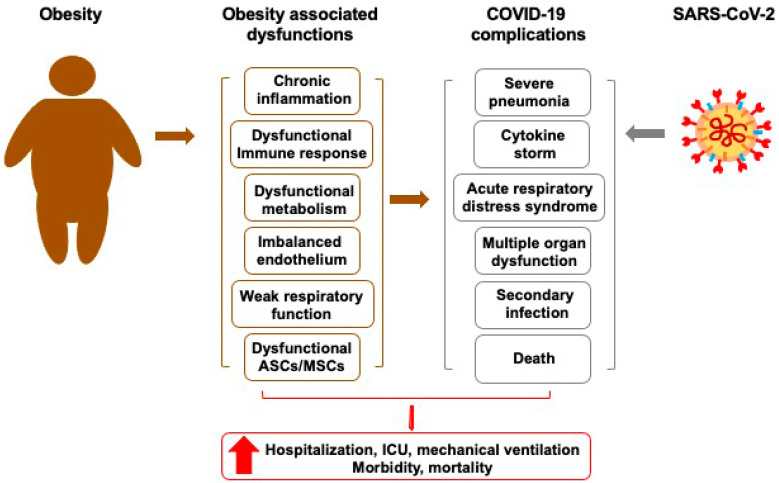
Schematic illustration of how obesity influence the development of COVID-19. Multiple pathological features of obesity, such as systematic chronic inflammation and dysfunctionality in ASCs/MSCs, immune response, metabolism, and endothelium, affect the progression and outcomes of COVID-19.

Various pathological molecular linkages have been proposed for the detrimental prognosis in obese COVID-19 patients (Figure 2) [51]. Adipose tissue (AT) in individuals with morbid obesity is characterized by low-grade inflammatory state altering innate and adaptive immunity [52,53]. AT consists of mature adipocytes and several types of stromal cells, including fibroblasts, adipose-derived mesenchymal stromal/stem cells (ASCs), endothelial cells, and various immune cells [54]. Morbid obesity has been shown to 1) compromise functions and properties of ASCs, changing their role from a pivotal regulator in AT to a threatening foe that induces hypoxia and secrets pro-inflammatory cytokines, and 2) lead to the loss of functional mesenchymal stem/stromal cells (MSCs) in various organs, including lung and brain [55,56]. Obese patients also often have impaired pulmonary function with reduced lung volumes and diaphragmatic strength, intensified airway resistance, and blunted gas exchange [57]. Accordingly, COVID-19 patients with obesity likely undergo a more serious respiratory damage [58]. Inversely, obese ASCs/MSCs adversely influence the immune response and further increase systemic inflammation [55]. 

AT expresses several receptors and enzymes for SARS-CoV-2 entry into and exit from cells, including the receptors ACE2, dipeptidyl peptidase 4 (DPP4), and cluster of differentiation (CD147), and one of the priming proteases, furin, which are upregulated in obese patients [59,60,61,62,63,64,65,66,67]. Consequently, diseased adipose tissues could be attacked by SARS-CoV-2, increasing inflammation/immune response and causing multi-organ failure [60]. 

A fine tuning of proinflammatory/pro-coagulant and anti-inflammatory/anti-coagulant states of the endothelium is disturbed by obesity-associated chronic inflammatory process, resulting in vascular endothelial dysfunction [68]. Importantly, ACE2 receptor and the priming proteases, furin and transmembrane protease serine 2 (TMPRSS2), are highly expressed in vascular endothelium [69]. Additionally, obesity-related inflammation and metabolic dysregulation lead to changes in nitric oxide (NO) and reactive oxygen species (ROS). Endothelial nitric oxide synthase (eNOS)-generated NO relaxes vascular smooth muscle cells, increases blood flow, and represses the release of mediators that recruit proinflammatory cell populations to the endothelium [70,71]; NO produced by inducible nitric oxide synthase (iNOS) in adipocytes and proinflammatory macrophages is toxic levels and increased in obesity [72]; ROS are often substantially raised in obesity and gives rise to perilous pathological changes [73]. Accordingly, the endothelium of patients with obesity are likely to be highly vulnerable to SARS-CoV-2 infection.

Multiple obesity-related factors activate the proinflammatory immune cells, including dendritic cells (DCs), M1 macrophages, T helper type 1 (Th1) cells, and CD8^+^ T cells [51]. Hypernutrition and saturated free fatty acids (FFAs) stimulate expression of TLRs in DCs, especially TLR2 and TLR4 that activate M1 macrophages [74]; activation of M1 macrophages outside of AT are further induced by upregulated secretion of AT inflammatory cytokines [75]. Additionally, elevated levels of lipopolysaccharide and adenosine triphosphate activate the NLRP3 inflammasome [76]. The secretion of crucial adipocytokines also increases the production of TNF-α, IL-6, and IL-12, resulting in primarily pathogenic proinflammatory Th1 cell population [77]. Unsurprisingly, the severity and mortality rate of obese individuals were increased in multiple infectious diseases, such as influenza [18,78]. Even after vaccination, individuals with obesity had a higher risk of influenza or influenza-like illnesses compared to healthy lean individuals [79]; their influenza specific antibody titer was dramatically reduced one year after vaccination [80,81]. Accordingly, diminished immune response provoked by obesity should be taken into account for developing SARS-CoV-2 vaccines as a key prophylactic approach. 

Importantly, one of the key hallmarks of obesity is adaptations in cardiac structure and function associated with a highly increased risk of cardiovascular diseases (CVD) [82]. Based on the previous coronavirus and influenza epidemics, pre-existing CVD and cardiovascular (CV) risk factors likely enhance vulnerability to COVID-19 [83,84,85,86,87,88,89,90]. Further, COVID-19 can worsen underlying CVD and cause de novo cardiac complications [91]. Several mechanisms are responsible for cardiovascular complications in COVID-19 [91,92,93]. SARS-CoV-2 can directly damage cardiomyocytes by binding to ACE2 and altering ACE2 signaling pathways with subsequent acute myocardial injury [92,93]. SARS-CoV-2 spreads through respiratory mucosa and concomitantly infect other cells, resulting in systemic inflammatory response and cytokine storm [94]. The systemic infection coupled with hypoxia can impair myocardial oxygen demand-supply relationship and lead to acute myocardial injury [91]. Additionally, systemic inflammation after infection can give rise to reduction in coronary blood flow (referred as acute coronary syndromes (ACS)), decrease in oxygen supply, destabilization of coronary plaque, and microthrombogenesis, which result in acute myocardial infarction [94,95,96]. Adverse effects of various therapies for COVID-19 and electrolyte imbalances can also have deleterious effects on the CV system [91]. In the case study of 187 patients with COVID-19, patients with underlying CVD, including hypertension, coronary heart disease, and cardiomyopathy, were more prone to experience myocardial injury during the course of COVID-19 [94]. Patients with underlying CVD with myocardial injury (demonstrated by escalation of troponin T (TnT) levels) had the highest mortality with the shortest survival term [94]. Interestingly, while patients with myocardial injury showed markedly increased mortality rate, patients with underlying CVD but with normal TnT levels experienced a more favorable prognosis [94]. These data suggest that myocardial biomarkers should be assessed in patients with CVD who develop COVID-19 for risk categorization and intervention. In the cohort study of 416 patients with confirmed COVID-19, patients with cardiac injury had higher mortality than those without cardiac injury; these patients had a history of coronary heart disease and hypertension, indicating that preexisting CV diseases were more susceptible COVID-19-induced heart injury [97]. Thus, the presence of pre-existing CV disease and/or development of acute cardiac injury are closely related to dramatically worse outcomes in COVID-19 patients.

### 2.3. Diabetes and COVID-19

The World Health Organization (WHO) estimates 422 million individuals in the world have diabetes with its prevalence rising rapidly; up to 95% of them suffer from T2DM. In the United States (US), 10.5% of the US population have been diagnosed with diabetes and 34.5% of the adult US population have prediabetes with the risk of onset of T2DM (National Diabetes Statistics Report, 2020). It is the unprecedented aging and obesity that are major contributors to the epidemic of T2DM. T2DM advances from prediabetes with impaired glucose tolerance (IGT) and/or MetS to overt diabetes with a diminution in functional β-cell mass [98,99]. Clinical studies have shown that decline in β-cell function derived from multiple risk factors, including obesity, inflammation, and IR, starts several years before the diagnosis of T2DM; fasting plasma glucose levels gradually increase for 10-12 years before diagnosis of T2DM [100]. At the time of the clinical onset of T2DM, patients have significantly fewer β-cells than healthy individuals as β-cells are metabolically afflicted and prone to apoptosis during the period of continual hyperglycemia [100]. 

T2DM is a pivotal predictor for COVID-19 severity and its complications [47]. Current data suggest worse outcomes in patients with preexisting diabetes [47]. A study in Hubei Province, China (a cohort of confirmed COVID-19 cases) showed that the fatality rate was higher in patients with T2DM compared to nondiabetic individuals (7.8% vs. 2.7%) [101]. Hyperglycemia is likely to modulate the risk of complications; the risk of death was lower in the subgroup with blood glucose (<7.5 (5.2–7.5) mmol/L; 1.1%) compared with poorly controlled blood glucose group (>7.6 (7.6–14.3) mmol/L; 11.0%) [101]. Similarly, analysis of the health data in the United Kingdom (17.4 million adults) revealed that uncontrolled diabetes was an independent risk for death from COVID-19 [50]. A multicenter observational study in France (diabetic patients hospitalized for COVID-19) also demonstrated that BMI was independently correlated with mechanical ventilation and/or death [102]. These studies suggest the connection between diabetes and COVID-19 mortality/complications.

Multiple mechanisms play roles in increasing the susceptibility of complications in diabetic COVID-19 patients [103]. Basically, as aforementioned, diabetes is tightly associated with the hallmarks and progression of obesity. Circulating levels of furin are elevated in patients with diabetes [104]. As observed in rodent models of diabetes [105,106], enhanced ACE2 expression in diverse tissues likely favors increased cellular binding and infection of SARS-CoV-2 [107]. Administration of insulin in diabetic mice decreased ACE2 protein expression in the lungs [105,106]. ACE2 is a part of the renin-angiotensin system (RAS) controlling blood pressure, balance of fluid and electrolyte, and systemic vascular resistance [25]. ACE catalyzes the conversion of the prohormone, angiotensin (Ang) I to the octapeptide, AngII, inducing vasoconstriction and proliferation; ACE2 converts AngII to Ang-(1–7), stimulating vasodilatation and suppressing cell growth [103]. Accordingly, suppressing ACE2 could hinder viral entry but may induce vasoconstriction and hypertension. Increased ratio of pulmonary ACE/ACE2 activity, which favors AngII generation, was observed in patients with ARDS [108]. Upon binding to ACE2, SARS-CoV was shown to downregulate ACE2 expression, leading to acute pulmonary damage via AngII [109]. Thus, in the setting of low ACE2 expression, upregulating ACE2 using agents that induce hypoglycemia and treat hypertension can be beneficial [103]. For an ACE2-targeted therapeutic strategy to prevent viral entry, infusion of human recombinant soluble ACE2 into the airway or bloodstream can be promising [110]. 

Altered immune response in diabetic patients is closely associated with a chronic low-level inflammation, lowered activity of NK cells, increased T cell activation, elevated inflammatory cytokine profile, and decreased Treg function, leading to dysregulated immune function in COVID-19 patients [55,111,112]. Microvascular endothelial dysfunction correlated with several key factors, including chronic inflammation, increased expression of iNOS, and elevated production of ROS, is also a frequent manifestation in diabetic patients [113]. Accordingly, dysfunctional shift of endothelial cells toward pro-thrombotic and pro-atherogenic states instigates severe impairment of COVID-19 on the cardiovascular system [51]. Diabetes, like obesity, is characterized by hypercoagulable state as well [114], featuring hypofibrinolysis and elevated levels of plasminogen activator inhibitor-1 (PAI-1) complement as well as increased platelet aggregation [115,116]. In patients with established T2DM, neutrophil extracellular traps (NETs), complexes of chromosomal DNA, histones, and granule proteins, were higher compared with healthy individuals [117,118]. Severely ill COVID-19 patients display coagulopathy/thrombosis [119], which is worsened by acute hyperglycemia in patients with diabetes [112]. Thus, optimal management of hyperglycemia is crucial for COVID-19 patients with diabetes [47,120]. 

## 3. Current Progresses in Prophylactic and Therapeutic Treatment Options for COVID-19 

### 3.1. Vaccination

Mass prophylactic vaccination is likely the only viable path to cost-effectively curb the COVID-19 pandemic. Following are some of the vaccine platforms that are being utilized for COVID-19 vaccine development in clinical trials and licensed in use on the market (Table 1) [121,122]. 

#### 3.1.1. Inactivated Vaccines

The virulent antigen or infectious viruses killed through physical or chemical processes are the basis of inactivated vaccines [121]. In case of SARS-CoV-2, the viruses are cultured in Vero cells and chemically inactivated [121]. Successful inactivated human pathogens as vaccines include Influenza, Hepatitis A and poliomyelitis [123,124,125]. The inactivated vaccines are safer to use and relatively easy to produce [126]; they express surface antigens that retain their epitope conformations to induce strong preventative humoral responses [127]. However, the reduced production of SARS-CoV-2 virus in cell culture and the requirement of biosafety level 3 (BSL3) facilities can lead to limited yield. The inactivated vaccines are commonly administered intramuscularly and adjuvanted with alum (aluminium hydroxide) [122]. Several inactivated vaccines against SARS-CoV-2 are currently being produced and some have entered in the clinical trials (Table 1). Examples of inactivated vaccine candidates include CoronaVac (Sinovac, Beijing, China) [128], BBIBP-CorV (Sinopharm, Shanghai, China) [129], Covaxin (Bharat Biotech, Hyderabad, India), and QazCovid-In (Research Institute for Biological Safety Problems, Gvardeysk, Kazakhstan), which are in clinical Phase III trials (Table 1).

#### 3.1.2. Live Attenuated Vaccines

Live attenuated vaccines use the live but a weakened version of the living virus [130], eliciting similar immune responses to natural infection without disease [131]. Generally, the viruses are exposed to unfavorable conditions (e.g., growth at lower temperature or in non-human cells) or genetically manipulated (e.g., modifying the viral genes through mutagenesis) [132,133,134,135,136]. Live attenuated seasonal influenza vaccine is an adequate example [137,138,139]. Important advantages of these vaccines are that they can be given intranasally, protecting the upper respiratory tract, which is the major portal of entry of SARS-CoV-2 [140,141,142]; they can induce both humoral and cellular immune responses. However, due to the live nature of the virus, this vaccine platform is less safe. Moreover, SARS-CoV-2 is novel coronavirus with a large (approximately 30,000 nucleotides) RNA, thus, it is challenging to generate genetically modified, live attenuated vaccine candidates [143]. Accordingly, only four live-attenuated vaccines are in preclinical trials; the one developed by Codagenix in collaboration with the Serum Institute of India is currently in clinical phase I. Intriguingly, “SARS-CoV-2/human/Korea/CNUHV03/2020” vaccine was generated by growing SARS-CoV-2 from 37 °C to 22 °C in Vero cells [144]. A single intranasal administration of this vaccine induced high titers of neutralizing antibodies and mucosal IgA antibodies as well as cellular immune responses in K18-hACE2 mice without detrimental systemic effects [144]. 

#### 3.1.3. Recombinant Protein Vaccines

Recombinant protein vaccines for SARS-CoV-2 can be classified into recombinant spike-protein-based vaccines, recombinant receptor binding domain (RBD)-based-vaccines, and virus-like particle (VLP)-based-vaccines, which carry no genome but display the spike ‘S’ protein on their surface [121,122]. A variety of eukaryotic expression systems are utilized to manufacture these recombinant proteins; even prokaryotic *E. coli* is used to express RBD [145,146,147]. These vaccines can be produced without handling live virus; considerable expertise has been acquired to produce recombinant subunit vaccines as shown in licensed FluBlok vaccine for influenza [148,149,150]. However, the spike ‘S’ protein is relatively hard to express, raising the subsequent questions of low yield and number of doses. While the RBD peptide may be easy to produce [151], the absence of other neutralizing epitopes present on the full-length S protein likely leads to the antigenic drift in RBD-based vaccines. Many recombinant protein vaccines are currently being produced and evaluated, including NVX-CoV2373 (Novavax, Gaithersburg, MD, USA; recombinant full-length S protein nanoparticle) [152] and ZF2001 (Anhui Zhifei Longcom Biopharmaceutical, Chongqing, China; RBD-dimer), which are in clinical Phase III trials (Table 1). 

#### 3.1.4. Replication-Incompetent/-Competent Vector Vaccines

Delivery vectors, such as viral vectors, are utilized to develop expression vector-based vaccines. Specifically, for viral vector-based vaccines, replication-incompetent or -competent viral backbone is engineered to express the target-pathogen-derived antigens.

The majority of the replication-incompetent vectors are based on recombinant adenovirus (AdV) vectors [153]; it is not necessary to handle live virus; considerable expertise has been acquired to produce large quantities in manufacturing the recombinant adenovirus vaccines; both B- and T-cell responses are induced by the vectors [121]. While pre-existing vector immunity affect some of these vectors, using adequate vector types, including adeno-associated viruses that do not induce much immunity by themselves, can overcome this complication [154]. Several replication-incompetent vector vaccines are in use, such as Ad26.COV2.S (Janssen Pharmaceutical Companies of Johnson & Johnson, Titusville, NJ, USA; authorized) [155], ChAdOx1 nCoV-19 (AstraZeneca, Cambridge, UK/University of Oxford, Oxford, UK; authorized) [156,157], Sputnik V (Gameleya Research Institute, Moscow, Russia; clinical phase III) [158], and Ad5-nCoV (CanSino Biologics, Tianjin, China; clinical phase III) [153,154] (Table 1). 

Replication-competent vectors are derived from attenuated viruses; to reduce the pathogenicity, the genes that are nonessential for viral replication in cultured cells in vitro are modified [159]. This approach induces more strong immunity owing to the vector’s capability to propagate in the vaccinated individuals [121]. Examples of replication-competent vector vaccines include DelNS1-2019-nCoV-RBD-OPT1 (Beijing Wantai Biological, Beijing, China; clinical phase II) and V591 (Institut Pasteur, Paris, France/Themis, Kenilworth, NJ, USA; clinical phase I/II). 

#### 3.1.5. DNA Vaccines

COVID-19 DNA vaccines are plasmid DNA encoding the SARS-CoV-2 spike ‘S’ gene under a mammalian promoter [121,122]. They can be produced at large scale in bacteria and offer high stability, whereas they often show low immunogenicity. Accordingly, booster doses and intracellular delivery devices, such as electroporators, are needed to attain the expected effect in vivo. There are several SARS-CoV-2 DNA vaccines currently in clinical trial stages, such as ZyCov-D (Zydus Cadila, Ahmedabad, India; clinical phase III), INO-4800 (Inovio Pharmaceuticals, Plymouth Meeting, PA, USA; clinical phase II/III) [160], and AG0301&AG0302 (AnGes, Osaka, Japan/Osaka University, Osaka, Japan/Takara Bio, Shiga, Japan; clinical phase II/III) (Table 1).

#### 3.1.6. RNA Vaccines

Two major types of RNA vaccines have been used against infectious pathogen: non-amplifying mRNA vaccines, either conventional or chemically modified base incorporated, and self-amplifying (or replicon) RNA vaccines retaining auto-replicative activity derived from a RNA virus vector [161]. Due to the self-replicative properties, self-amplifying RNA vaccines require a lower dose of RNA than non-amplifying mRNA vaccines [162]. RNA vaccines have a key advantage compared to DNA vaccines that require an additional transcription step in in vivo systems; RNA vaccines can be easily synthesized in vitro in the lab. Furthermore, RNA vaccines reduce the risk of insertional mutagenesis. However, RNA vaccines require frozen storage due to their thermolabile nature (unstable at high temperatures). Consequently, modifications can be introduced for their safety, stability, and immunogenicity [163]. Lipid nanoparticle (LNP) platform has been utilized for RNA vaccine delivery [164,165]. There are several SARS-CoV-2 RNA-vaccines in clinical trials or licensed for vaccination on the market, including BNT162b2 (Pfizer, NY, NY, USA/BioNTech, Mainz, Germany; authorized) [166], mRNA-1273 (Moderna, Cambridge, MA, USA/NIAID, Rockville, MD, USA; authorized) [167], CVnCoV (CureVac, Tübingen, Germany; clinical phase III) (Table 1). Specifically, Pfizer’s BNT162b2 RNA vaccine is a LNP-formulated, nucleoside-modified (two proline mutations) RNA vaccine that encodes a prefusion stabilized, membrane-anchored SARS-CoV-2 full-length spike protein [166]. A report from a clinical trial (ClinicalTrials.gov number, NCT04368728; accessed 04/10/2021) demonstrated that BNT162b2 was 95% effective in preventing Covid-19; over 90% vaccine efficacy was observed even in the presence of coexisting conditions (e.g., obesity) [166]. mRNA-1273 from Moderna encodes the full-length spike ‘S’ protein with two stabilizing mutations and is encapsulated in LNPs for delivery. Phase III clinical trials (ClinicalTrials.gov number, NCT04470427; accessed 04/10/2021) with geriatric patients demonstrated that mRNA-1273 showed 95% efficacy across all demographics [168]. 

### 3.2. Antivirals and Therapeutics

There are three general methods to search for the promising antivirals and therapeutic drugs of human SARS-CoV-2: utilization of the current broad-spectrum drugs, testing chemical libraries with existing compounds or databases, and development of new specific drugs based on the genome and biophysical understanding of SARS-CoV-2 [169]. Following are some of the antivirals and therapeutics for treating COVID-19 in clinical trials and in use (Table 2) [170,171].

#### 3.2.1. Antivirals

##### Remdesivir

Remdesivir (RDV) is one of the leading antivirals against SARS-CoV-2. While it was developed for treating Ebola virus [172], it displays the activity against other RNA viruses, such as paramyxoviruses, pneumoviruses, and coronaviruses (e.g., SARS-CoV and MERS-CoV) [173]. RDV, also known as GS-5734, is a 1′-cyano-substituted adenosine nucleotide analog [174]. As a prodrug, RDV is metabolized in cells and tissues to an active nucleoside triphosphate (RDV-TP; GS-443902) that inhibits viral RNA-dependent RNA polymerases (RdRps) by incorporating RDV-TP into nascent RNA chains [175,176]. The Adaptive COVID-19 Treatment Trial (ACTT; NCT04280705) showed that RDV was more efficient than a placebo in treating hospitalized COVID-19 patients [177]. RDV treatment shortened the time to recovery (11 days versus 15 days) and reduced respiratory infection as well as mortality compared to placebo [177]. RDV was approved by the US Food and Drug Administration (FDA) for treating adults and pediatric patients requiring hospitalization for COVID-19; it is administered via intravenous (IV) infusion [171]. There are several ongoing clinical trials with RDV, including inhaled RDV formulation for early-stage COVID-19 [170].

##### Favipiravir

Favipiravir is an antiviral approved for treating influenza in Japan; it exhibits antiviral activity against several other viruses, including chikungunya virus, Rift Valley fever virus, hepatitis C virus (HCV), and the West Nile virus by inhibiting RdRp [178,179,180,181,182]. In an open-label control study in China, favipiravir treatment showed better therapeutic responses on COVID-19 with regard to disease progression and SARS-CoV-2 clearance [183]; other trials have reported that favipiravir alleviated some of the COVID-19 symptoms, such as cough, and stimulated defervescence [184]. These studies suggest the potential beneficial effects of favipiravir on patients with mild COVID-19. There are various ongoing clinical trials with favipiravir; it has been approved for use in multiple countries.

##### Lopinavir

Lopinavir (LPV) is a human immunodeficiency virus (HIV)-1 protease inhibitor administered in fixed-dose combination with ritonavir (LPV/r). Most of the protease inhibitory effects are attributed to LPV, while ritonavir enhances the pharmacokinetic and pharmacodynamic properties of LPV [185]. SARS-CoV, MERS CoV, and SARS-CoV-2 coronaviruses are RNA viruses like HIV; the nonstructural protein of coronaviruses, especially the main protease 3C-like protease (3CLpro), is involved in the proteolysis of the replicase polyprotein, which is essential for viral infection [186]. As shown in treating patients in South Korea and in China in early SARS-CoV-2 outbreak, LPV/r was expected to be beneficial for COVID-19 treatment based on the sequence similarity of the CoV proteases [187,188]. However, the results of the Efficacy of Lopinavir Plus Ritonavir and Arbidol Against Novel Coronavirus Infection (ELACOI) and Randomized Evaluation of COVID-19 Therapy (RECOVERY) as well as WHO’s Solidarity trial showed little or no benefit of LPV/r to hospitalized COVID-19 patients [189,190,191]. Several clinical trials are currently exploring the effects of LPV/r as prophylaxis or for early-stage COVID-19 [170]. 

##### Ribavirin

Ribavirin is a guanosine analog that interferes with viral RNA synthesis by inhibiting viral RdRp and mRNA capping [192]. In primate models, using rhesus macaques, ribavirin alone or in combination with interferon (IFN)-α2b reduced virus replication of MERS-CoV and ameliorated the clinical outcomes with moderation of the host response [193]. Consistent with the positive clinical outcomes in some MERS cases using the combination of ribavirin with LPV/r and IFNs [194,195], the combination of ribavirin with IFN-β1b and LPV/r alleviated symptoms in patients with mild to moderate COVID-19 [196]. Contrarily, combinations of ribavirin + IFN-α, LPV/r + IFN-α, and ribavirin + LPV/r + IFN-α, or combination of ribavirin + LPV/r showed no significantly different antiviral effects or even adverse effects in a randomized open-labeled prospective study with mild to moderate COVID-19 patients [197]. Ongoing clinical trials are underway to determine the potential of ribavirin in COVID-19 treatment [170]. 

##### Umifenovir 

Umifenovir (brand name Arbidol) is an antiviral approved in China and Russia for treating influenza. It binds in a hydrophobic cavity in the hemagglutinin (HA) trimer stem, inhibiting viral membrane fusion in the low pH of the endosome [198]. A retrospective study suggested that umifenovir treatment increased the discharging rate with a decrease in the mortality rate of COVID-19 patients [199]; the results of a randomized, controlled trial demonstrated that treatment of hydroxychloroquine (HCQ) + umifenovir resulted in substantially shorter duration of hospitalization and decreased intensive care unit (ICU) admission rates than HCQ + LPV/r treatment in hospitalized COVID-19 patients [200]. However, another retrospective study demonstrated little effect of umifenovir on improving clinical consequences in non-ICU patients [201], suggesting more trials for accessing the effects of umifenovir in COVID-19. 

##### Chloroquine and Hydroxychloroquine

Chloroquine (CQ) and hydroxychloroquine (HCQ) are used to prevent/treat malaria. CQ has been shown to inhibit the replication of different CoVs, including SARS-CoV, MERS-CoV, and SARS-CoV-2 among others; HCQ was shown to have potent in vitro inhibitory effects against SARS-CoV-2 replication [202]. CQ and HCQ increase endosomal pH, thus inhibiting virus-endosome fusion for viral endocytosis [203,204]. An open-label non-randomized clinical trial for HCQ with azithromycin, which was shown to be active in vitro against Zika and Ebola viruses and prevent severe respiratory tract infections [205,206,207], demonstrated that HCQ significantly reduced viral load in COVID-19 patients; its effect was reinforced by azithromycin [208]. However, the final results of the RECOVERY groups HCQ trial showed that hospitalized COVID-19 patients receiving HCQ showed similar mortality rate to the patients without treatment [209]. Several trials are still ongoing to determine the effects of CQ and HCQ as prophylaxis or for early-stage COVID-19 [170].

#### 3.2.2. Immune-Based Therapeutics

##### Neutralizing Antibodies

Convalescent plasma (CP) from individuals following resolution of infection contains neutralizing Abs (NAbs) that bind to the pathogen and neutralize its biological effect [210]. Accordingly, plasma exchange can be a favorable option for treating COVID-19 patients. While a clinical trial from eastern Anatolia showed that patients with early-stage COVID-19 who did not need mechanical ventilation improved with CP treatment [211], the largest completed CP study to date (PlasmAr; ClinicalTrials.gov number, NCT04383535; accessed 04/10/2021) indicated no significant differences in clinical status or overall mortality between COVID-19 patients with severe pneumonia treated with CP and those who received placebo [212]. Furthermore, considering the potential risks arising from CP therapy, including viral or bacterial infections, lung damage, and allergic reactions [213], the use of mAbs and mAb cocktails have been presented. Several studies demonstrated NAbs that can be used for COVID-19 treatment by targeting diverse epitopes on the spike ‘S’ protein [214,215,216,217]. Administration of LY-CoV555 (bamlanivimab; granted emergency use authorization by the US-FDA), an IgG1targeting the SARS-CoV-2 ‘S’ protein, into the patients diagnosed mild or moderate CoVID-19 resulted in accelerating the natural decline in viral load over time [218]. The REGN-COV2 (granted emergency use authorization by the US-FDA), an antibody cocktail of two anti-SARS-CoV-2 ‘S’ protein Abs (REGN10933 + REGN10987), diminished viral load [219].

##### Cytokine Inhibitors: Tocilizumab 

The primary cause of death in seriously ill COVID-19 patients is ARDS and multi-organ system failure [171]. The heightened synthesis of inflammatory cytokines/chemokines leading to cytokine release syndrome (CRS) has been implicated as a main culprit; IL-6 is a key inflammatory driver, thus, can be a biomarker indicating worsening of COVID-19 [220,221]. Tocilizumab is a recombinant humanized monoclonal antibody that competitively inhibits the binding of IL-6 to soluble and membrane-bound IL-6 receptor (IL-6R) [222]. It has been approved by the FDA mainly for treating rheumatoid arthritis (RA) [222]. Several studies demonstrated that tocilizumab therapy reduced ICU admissions and mortality in critical COVID-19 patients; treatment with low-dose tocilizumab in non-critical hospitalized COVID-19 patients alleviated inflammation with faster defervescence [223,224,225,226,227,228]. However, contradicting results with no reduction in mortality and progression to mechanical ventilation were observed in trials for tocilizumab [229,230]. These contradictory results underscore that larger placebo-controlled studies are required to determine the effects of tocilizumab in COVID-19.

##### JAK Inhibitors: Baricitinib

Four mammalian Janus kinases (JAKs), JAK1, JAK2, JAK3, and tyrosine kinase 2 (Tyk2) are protein tyrosine kinases [231]. The JAK/signal transducer and activator of transcription (STAT) pathway plays important roles in cytokine-/interferon-induced inflammatory responses, thus, JAK inhibition can be a potential resource for treating COVID-19 [232]. Baricitinib is a JAK1/2 inhibitor licensed for the treatment of RA [233]; it has anti-viral effects by its affinity for adaptor-associated protein kinase 1 (AAK1), an adaptor protein-2 complex (AP2)-associated protein, thus, reducing SARS-CoV-2 endocytosis [234]. Baricitinib-therapy started in the early phase of COVID-19 reduced the fatality rate and ICU admission rate with decreased SARS-CoV-2 viral burden without serious adverse events [235,236]. A double-blind, randomized, placebo-controlled trial for baricitinib plus RDV reported that the combination reduced recovery time and accelerated improvement in clinical status of hospitalized COVID-19 patients, notably those under high-flow oxygen or noninvasive ventilation support [237]. Several trials to evaluate the benefits of baricitinib in treating moderate and severe COVID-19 are underway [170].

#### 3.2.3. Statin Therapy

Statins reduce the synthesis of cholesterol in the liver by competitively repressing the hydroxymethyl-glutaryl coenzyme A (HMG-CoA) reductase [238]. Therefore, statins have been widely used to prevent and treat CVD as obesity-induced dyslipidemia is one of traditional CV risk factors [239]. Statins have also demonstrated inhibitory effects on several viruses, including influenza [240]. The role of statins in treating COVID-19 has been proposed as their cholesterol-lowering capacity can destabilize the formation of lipid rafts that are needed for ACE2-mediated SARS-CoV-2 infection [241,242]. Moreover, upon entering the cell, SARS-CoV-2 downregulates expression of ACE2, thus reducing its protective effects on various tissues and leading to organ injury; statins have been shown to increase the expression of ACE2 in animal models [243,244,245,246,247]. Statins also have pleiotropic effects: restoring vascular redox balance, inhibiting the thrombogenic response, and decreasing oxidative stress and inflammation by inhibiting the MYD88-NF-κB proinflammatory pathway [243,248,249]. Intriguingly, increased secretion of anti-inflammatory cytokines, such as IL-2 and IL-10, seems to represent a distinctive feature of COVID-19 [95]. While the dysregulation of secreted pro- and anti-inflammatory cytokines likely plays a critical role in the progression of ARDS and the severity of the diagnosis, cholesterol biosynthesis pathway was shown to be essential for dictating the CD4^+^ T cell switch from the effector (Th1 cell) to the anti-inflammatory (IL-10-secreting Th2 cell) phenotype [249,250]. A retrospective study on 13,981 patients with COVID-19 in Hubei Province, China reported that in-hospital use of statins compared to non-statin use was significantly associated with a lower risk of death and a less inflammatory response during the entire hospitalization period [251]. Another retrospective single-center study (University of California San Diego Health) showed that statin use during the 30 days prior to admission for COVID-19 was associated with a lower risk of developing severe COVID-19, and a faster time to recovery among patients without severe disease [252]. However, an observational multicenter study with 842 COVID-19 patients showed that statin therapy was associated with a worse disease severity (but nevertheless, not with in-hospital mortality) [253]. Thus, statin use can rather be considered as a proxy of underlying comorbidities; in patients on high-intensity statin treatment, the enhanced severity of underlying CV risk factors and CVD can explain the higher severity of COVID-19 [253]. Accordingly, further prospective studies and randomized controlled clinical trials are needed to more definitely determine the overall clinical benefits of statin treatment for COVID-19-related pathologies.

#### 3.2.4. Anticoagulation Therapy

Many severe COVID-19 patients show coagulation abnormalities with an increased risk of death, indicating the presence of a hypercoagulable state also detected in obesity and diabetes [114,254]. The coagulopathy associated with COVID-19 is a combination of low-grade DIC (mixture of thrombocytopenia, prolonged prothrombin time, and increased D-dimer) and confined pulmonary thrombotic microangiopathy, which can result in organ dysfunction in the most severely affected patients [254]. Several distinctive pulmonary vascular pathological features of COVID-19 are 1) severe endothelial injury associated with disrupted endothelial cell membranes, 2) widespread vascular thrombosis with microangiopathy, 3) occlusion of alveolar capillaries, and 4) substantial new vessel growth via intussusceptive angiogenesis [255,256]. Severe COVID-19 is also associated with increased concentrations of proinflammatory cytokines [95]. Specifically, IL-6 can induce tissue factor expression on mononuclear cells, which initiates coagulation activation and thrombin generation; TNF-α and IL-1 mediate a suppression of endogenous anticoagulant pathways [254]. A retrospective study in China (including 449 patients admitted to hospital with severe COVID-19 infection) showed a lower mortality in patients with COVID-19-associated coagulopathy who received prophylactic heparin than in patients not receiving anticoagulant treatment [257]. Particularly, in patients with increased concentrations of d-dimer (6 times the upper limit of normal), mortality was lower in heparin-treated patients than those not treated with heparin, while a prospective randomized controlled trial is needed to confirm these results [257]. Another study with 844 COVID-19 patients reported that oral anticoagulants (OACs) appeared to be ineffective in reducing mortality rate, whereas heparin resulted to be an effective treatment when lung disease was severe; larger studies are needed to confirm these findings [258]. Heparin inhibits cellular invasion by SARS-CoV-2 by binding to the spike S1 protein receptor binding domain and inducing a conformational change; it also exhibits anti-inflammatory effects, inhibiting inflammatory cell infiltration and dampening pro-inflammatory signals; these features may be critical in determining different outcomes compared to OACs [259,260]. Hence, the benefits of heparin treatment should be continuously evaluated, while considering the risk factors, including the increased likelihood of bleeding and other contraindications for anticoagulation.

## 4. Conclusions

SARS-CoV-2 caused a grievous pandemic of COVID-19. Accordingly, multiple clinical trials are in progress, evaluating treatment options for COVID-19, some of which have been listed in this review (Table 1 and Table 2). Vaccination could be the most effective prophylactic measure to prevent the ongoing COVID-19 pandemic as seen in continuing global vaccination programs with licensed COVID-19 vaccines (Table 1). Antivirals, including RDV and favipiravir, and NAbs, such as LY-CoV555 and REGN-COV2, have been successfully applied to treat the patients with COVID-19 whereas more trials are needed to adequately evaluate their effects. The severity of COVID-19 is strongly associated with age; there is an increased prevalence of MetS, such as obesity and diabetes, in people hospitalized with severe COVID-19 illness. Notably, the mRNA-1273 and BNT162b2 vaccines were equally protective in elderly people and individuals with obesity/T2DM [166,168]. Nevertheless, the long-term durability of immunogenicity has not been assessed; both the occurrence of adverse events after the second dose and more apprehensive information on the duration of protective immunity remain to be determined. Furthermore, comprehensive understanding of the underlying mechanisms of the adverse outcomes of COVID-19 illness in elderly people and hospitalized people with MetS will give insight into the development of disease-specific therapeutic interventions.

## 5. Future Research Directions

### 5.1. Therapeutic Perspectives

Data from retrospective observations indicate that glycemia optimization treatment likely reduces the risk of severe COVID-19 [261]. While GLP-1 receptor (GLP-1R) agonists are approved for weight loss, scant information is obtainable whether this class of drugs is reliable and beneficial in SARS-CoV-2-infected people with obesity, needless to say the necessity of the safety profiles of other glucose-lowering agents in people with clinical symptoms of COVID-19. Pre-clinical studies demonstrate that treatment of GLP-1R agonists attenuated virus-triggered pulmonary inflammation in mice [262,263,264], whereas data of how GLP-1R agonists function in experimental or clinical SARS-CoV-2 infection are not yet available. In addition, whether the risk of severe COVID-19 infection can be dampened by weight loss has not been systemically studied. Intriguingly, treatment of obese mice with metformin, not weight loss, was able to improve survival to influenza in obesity, suggesting an importance of pharmaceutical interventions [265].

Individuals with obesity and/or obesity-derived T2DM with prolonged immobility in the hospital are vulnerable for thromboembolic disease [261]. As shown in severely ill, hospitalized people with COVID-19 in Wuhan, China [266], one of the common pathophysiology for greater COVID-19 vulnerability in people with obesity and/or T2DM is their predisposition for developing coagulation-related complications. Consequently, the forthcoming results of various anti-coagulant strategies can be critical therapeutic options for preventing SARS-CoV-2-related coagulopathy.

### 5.2. Mechanistic Perspectives 

The details of how much inflammation occurs and gene expression is dysregulated in metabolically important tissues are not readily available in active SARS-CoV-2 infection. While inflammatory responses in lung, brain, heart, kidney, and vascular tissues have been an intense focus of autopsy analyses [267], studies of the adipose tissues, pancreas, or endocrine organs are very limited. Furthermore, little molecular data is at hand from tissue biopsies of SARS-CoV-2-infected people with obesity and diabetes. Major conclusions regarding the mechanisms of viral entry and the features of glucose-lowering drugs are stemmed from preclinical studies or people without COVID-19 [268], thus, may be irrelevant for understanding human SARS-CoV-2 infection.

SARS-CoV-2 is incapable to propagate in wild-type mice owing to incompatibility of the spike ‘S’ protein with mouse ACE2 [269]. Thus, transgenic mice expressing human ACE2 in proper lung epithelial cells were established to model the replication and susceptibility of SARS-CoV-2 in vivo [270,271,272]. Recently, mouse-adapted SARS-CoV-2 (SARS-CoV-2 MA) was generated [273,274]. The SARS-CoV-2 MA model exhibited more clinically applicable phenotypes than those seen in HFH4-hACE2 transgenic mice expressing human ACE2 under the control of a lung ciliated epithelial cell-specific HFH4/FOXJ1 promoter [273]. Consequently, SARS-CoV-2 MA can be applied to the existing mouse models to better understand the impact of COVID-19 on obesity, such as genetically obese mouse models, including leptin-deficient (*ob/ob*) and leptin-receptor-deficient (*db/db*), as well as high-fat diet-induced-obese (DIO) mouse model [275,276,277]. Significant scientific gaps exist in the roles and mechanisms of host genetics and aging/MetS governing SARS-CoV-2 pathogenesis. Moreover, the detailed protective or pathogenic immune responses remain elusive, relating to COVID-19 severity. It is therefore of paramount importance to evaluate the performance of vaccines and therapeutics in the most vulnerable populations of SARS-CoV-2 infection.

## Figures and Tables

**Table 1:** COVID-19 vaccines currently in clinical trials or authorized.

Company/Organization	Brand Name	Vaccine Type/Platform	Clinical Trial Status
**Inactivated/Killed Vaccines**			
Bharat Biotech, Indian Council of Medical Research, National Institute of Virology, Ocugen, Precisa Medicamentos	Covaxin	Inactivated	III
Institute of Medical Biology, Chinese Academy of Medical Sciences	COVID-19 vaccine	Inactivated	III
Sinovac, Instituto Butantan, Bio Farma	CoronaVac (PiCoVacc)	Inactivated	III
Beijing Institute of Biological Products, Sinopharm	BBIBP-CorV	Inactivated	III
Wuhan Institute of Biological Products, Sinopharm	COVID-19 vaccine	Inactivated	III
Research Institute for Biological Safety Problems, Republic of Kazakhstan	QazCovid-In	Inactivated	III
**Non-Replicating or Replicating Viral Vector Vaccines**			
CanSino Biologics, Beijing Institute of Biotechnology, Petrovax	Ad5-nCoV (Convidencia)	Non-replicating viral vector	III
AstraZeneca, University of Oxford, Serum Institute of India	ChAdOx1 nCoV-19 (AZD1222)	Non-replicating viral vector	Authorized
Gameleya Research Institute	Sputnik V (Gam-COVID-Vac)	Non-replicating viral vector	III
Janssen Pharmaceutical Companies of Johnson & Johnson	Ad26.COV2.S (JNJ-78436725)	Non-replicating viral vector	Authorized
**DNA-Based Vaccines**			
AnGes, Osaka University, Takara Bio	AG0301&AG0302	Plasmid	II/III
Inovio Pharmaceuticals, International Vaccine Institute	INO-4800	Plasmid	II/III
Zydus Cadila	ZyCov-D	Plasmid	III
**RNA-Based Vaccines**			
CureVac	CVnCoV	LNP-mRNA	III
Moderna, NIAID (VRC)	mRNA-1273	LNP-mRNA	Authorized
Pfizer, BioNTech, Fosun Pharma	BNT162b2	LNP-mRNA	Authorized
**Protein Subunit Vaccines**			
Novavax	NVX-CoV2373	Full length recombinant SARS-COV-2 glycoprotein nanoparticle vaccine adjuvanted with Matrix M	III
Anhui Zhifei Longcom Biopharmaceutical, Chinese Academy of Sciences	ZF2001	Adjuvanted recombinant protein (RBD-Dimer)	III
Instituto Finlay de Vacunas	FINLAY-FR-2	rRBD produced in CHO-cell chemically conjugate to tetanus toxoid	III

**Table 2:** Antiviral and therapeutic drugs for treating COVID-19.

Drug	Target/Mode of Action	Status
Remdesivir (RDV)	Adenosine analog viral RdRp inhibitor	Effects at middle COVID-19 stage demonstrated; Approved by the US-FDA
Favipiravir	Influenza virus emergency drug (Japan) Purine analogViral RdRp inhibitor	Effects at early COVID-19 stage demonstrated; EUA (outside USA)
Lopinavir + ritonavir	HIV protease inhibitor (approved) Potential CoV protease inhibitor	Effects at early COVID-19 stage indicated
Ribavirin	Guanosine analog Inhibits GTP synthesis Viral mutagenesis Immunomodulatory activity	Effects at early COVID-19 stage indicated
Umifenovir	Influenza treatment and prophylaxis (China and Japan) Viral endocytosis inhibitor Inhibitor of viral genome replication	Effects at early COVID-19 stage indicated
Chloroquine (CQ), hydroxychloroquine (HCQ)	Anti-malaria Anti-rheumatoid arthritis (HCQ) Viral endocytosis inhibitor in vitro	Effects at early COVID-19 stage indicated
NAbs: LY-CoV555, REGN-COV2	Viral neutralization	Effects at early COVID-19 stage demonstrated; EUA (USA)
Tocilizumab	IL-6 receptor	Effects at middle to late COVID-19 stage demonstrated
Baricitinib	JAK1/2	Effects at middle to late COVID-19 stage indicated

Early COVID-19 stage, first week of infection, viral phase, pre-/early symptomatic phase; middle COVID-9 stage, second week of infection, symptomatic, early stages of hyperinflammation; late COVID-19 stage, beyond second week of infection, hyperinflammatory to thrombotic stages. EUA: Emergency use authorization.

## Data Availability

Not applicable.

## Abbreviations

COVID-19	Coronavirus disease 2019
SARS-CoV-2	Severe acute respiratory syndrome coronavirus 2
T2DM	Type 2 diabetes mellitus
ACE2	Angiotensin-converting enzyme 2
ARDS	Acute respiratory distress syndrome
DM	Diabetes mellitus
SASP	Senescence-associated secretory phenotype
NK	Natural killer
MetS	Metabolic syndrome
IR	Insulin resistance
HDL	High-density lipoprotein
IFR	Infection fatality ratio
DIC	Disseminated intervascular coagulation
AMs	Alveolar macrophages
TLR	Toll-like receptor
PD-1	Programmed cell death-1
IL	Interleukin
TNF	Tumor necrosis factor
CRP	C-reactive protein
NAD	Nicotinamide adenine dinucleotide
NLRP3	Nucleotide-binding domain (NOD)-like receptor protein 3
PARPs	Poly-ADP-ribose polymerases
AGEs	Advanced glycation end products
AMPK	5′-AMP-activated protein kinase
BMI	Body mass index
AT	Adipose tissue
ASCs	Adipose-derived mesenchymal stromal/stem cells
MSCs	Mesenchymal stem/stromal cells
DPP4	Dipeptidyl peptidase 4
CD147	Cluster of differentiation
TMPRSS2	Transmembrane protease serine 2
NO	Nitric oxide
ROS	Reactive oxygen species
eNOS	Endothelial nitric oxide synthase
iNOS	Inducible nitric oxide synthase
DCs	Dendritic cells
Th1	T helper type 1
FFAs	Free fatty acids
CVD	Cardiovascular diseases
CV	Cardiovascular
ACS	Acute coronary syndromes
WHO	World Health Organization
US	United States
IGT	Impaired glucose tolerance
RAS	Renin-angiotensin system
Ang	Angiotensin
GLP-1	Glucagon-like peptide-1
TZDs	Thiazolidinediones
NETs	Neutrophil extracellular traps
BSL3	Biosafety level 3
RBD	Receptor binding domain
VLP	Virus-like particle
AdV	Adenovirus
LNP	Lipid nanoparticle
RDV	Remdesivir
RdRps	RNA-dependent RNA polymerases
FDA	Food and Drug Administration
IV	Intravenous
HCV	Hepatitis C virus
LPV	Lopinavir
HIV	Human immunodeficiency virus
CYP3A4	Cytochrome P450 3A4
3CLpro	3C-like protease
ELACOI	Efficacy of Lopinavir Plus Ritonavir and Arbidol Against Novel Coronavirus Infection
RECOVERY	Randomized Evaluation of COVID-19 Therapy
IFN	Interferon
HA	Hemagglutinin
HCQ	Hydroxychloroquine
CQ	Chloroquine
CP	Convalescent plasma
NAbs	Neutralizing Abs
CRS	Cytokine release syndrome
IL-6R	IL-6 receptor
JAK	Janus kinase
Tyk2	Tyrosine kinase 2
STAT	Signal transducer and activator of transcription
RA	Rheumatoid arthritis
AAK1	Adaptor-associated protein kinase 1
AP2	Adaptor protein-2 complex
HMG-CoA	Hydroxymethyl-glutaryl coenzyme A
OACs	Oral anticoagulants
GLP-1R	GLP-1 receptor
SARS-CoV-2 MA	Mouse-adapted SARS-CoV-2
DIO	Diet-induced-obese
